# Inter- and intra-observer reliability of the “Assessment of Motor Repertoire- 3 to 5 Months” based on video recordings of infants with Prader-Willi syndrome

**DOI:** 10.1186/s12887-022-03224-2

**Published:** 2022-03-22

**Authors:** Jun Wang, Xiushu Shen, Hong Yang, Wei Shi, Xiaoyun Zhu, Herong Gao, Huanhuan Yin, Fanzhe Meng, Yun Wu

**Affiliations:** 1 Department of Rehabilitation, Children’s Hospital, Fudan University, Shanghai, 201102 China; 2 Key Laboratory of Neonatal Disease, Ministry of Health, Shanghai, 201102 China

**Keywords:** Inter- and intra-observer reliability, General movements assessment, Infants, Prader-willi syndrome

## Abstract

**Background:**

The “Assessment of Motor Repertoire—3 to 5 Months”, which is a part of Prechtl's General Movements Assessment (GMA), has been gradually applied to infants with genetic metabolic disorders. However, there have been no studies on the application of the GMA for infants with Prader-Willi syndrome (PWS).

**Aims:**

The purpose of this study was to determine the inter- and intra-observer reliability of the assessment tool in a population of infants with PWS.

**Study design:**

This was a reliability and agreement study.

**Subjects:**

This was a cross-sectional study with15 infants with PWS born at an average gestational age of 38 weeks.

**Outcome measures:**

Standardized video recordings of 15 infants with PWS (corrected ages of 3 to 5 months) were independently assessed by three observers. Kappa and ICC statistics were applied in inter- and intra- observer reliability analyses.

**Results:**

The overall reliability ICC values of the “Motor Optimality Score” (MOS) ranged from 0.84 to 0.98, and the pairwise agreement ranged between 0.86 and 0.95 for inter- observe reliability. In addition, ICC values for the MOS ranged between 0.95 and 0.98 for tester agreement in intra-observer reliability.

Complete agreement reliability (100%) was achieved in the subcategories of “Fidgety Movements” and “Movement Character” for the inter- and intra-observer reliability. Moderate to high inter- and intra-observer reliability were found in the subcategories of “Repertoire of Co-Existent Other Movements”, “Quality of Other Movements” and “Posture”, with kappa values ranging between 0.63 and 1.00.

**Conclusion:**

There were high levels of inter-and intra-observer agreement in the “Assessment of Motor Repertoire—3 to 5 Months” for infants with PWS. It is possible to carry out standardized quantitative assessments of the motor performance of infants with PWS.

## Introduction

Prader–Willi syndrome (PWS) is a disorder that is characterized by a genetic imprinting defect associated with chromosome 15q11-13 [1]. In western countries, the estimated prevalence of PWS in different populations ranges from 1/10000 to 1/30000, while there is a lack of epidemiological data in China [2–5]. The phenotype of PWS gradually emerges with development. The main characteristics of PWS include a variety of physical, cognitive, and behavioural defects [6]. The most marked features are hypotonia in infancy, hypogonadism, short stature, obesity, developmental delays, and intellectual impairments [1]. The occurrence of symptoms is mostly related to age; not all clinical phenotypes are expressed in all patients, and the severity of disability varies among patients [6]. Meanwhile, PWS is one of the important causes of symptomatic morbid obesity, and early diagnosis and reasonable intervention are crucial to improve the quality of life of these children, prevent serious complications and prolong life [2–5, 7, 8].

Therefore, it is of great clinical significance to identify any abnormalities in motor performance as early as possible, particularly in infants with PWS, before genetic diagnosis has been confirmed to provide timely early intervention.

Prechtl's General Movements Assessment (GMA) is a safe, valid, reliable and noninvasive assessment tool for identifying infants at risk of poor neurodevelopmental outcomes, particularly cerebral palsy, with the aim of intervening early and improving outcomes [9, 10]. Initially, the assessment of general movements(GMs)was mainly used to evaluate infants with brain injury. Recently, it has been gradually applied to infants with certain genetic and metabolic diseases [10–15].

GMs were divided into two stages according to the development process: the writhing movement stage (writhing stage) and the fidgety movements stage (fidgety stage). GM assessment includes an overall assessment and a detailed assessment. The motor optimality score (MOS) of the “Assessment of Motor Repertoire—3 to 5 Months” in a detailed assessment during the fidgety movements stage can be used not only to quantify the efficacy of early interventions but also to quantitatively analyse the relation between the data of the motor repertoire for an infant and the data obtained from follow-up studies [15–18].

The researchers conducted a reliability study of the “Assessment of Motor Repertoire -3 to 5 Months”, and the study results showed that the inter-observer reliability of the assessment of the total MOS for infants with risk factors was very high, as the intraclass correlation coefficient (ICC) was 0.87, and the ICCs for the pairwise analyses ranged between 0.80 and 0.94 [19]. Based on the results of the reliability study of the MOS scale, Dafne Herrero explored the relationship between the MOS of the repertoire at 3- to 5-months of age for infants with Down syndrome and their eventual motor performance. The MOS of infants with Down syndrome was higher than that of infants who were later diagnosed with cerebral palsy, but lower than that of infants with normal neurological outcomes [15].

However, until now, there have been no studies on the application of GMs in the early assessment and prediction of motor developmental outcomes for infants with PWS. In addition, sufficient inter-and intra-observer reliability is required for different testers to correctly use an instrument for scientific and clinical purposes.

Therefore, the purpose of this study was to determine the inter- and intra-observer reliability of the “Motor Repertoire -3 to 5 Months” assessment tool based on video recordings of infants with PWS.

## Methods

The procedures and the reporting of this study was carried out in accordance with the Guidelines for Reporting Reliability and Agreement Studies (GRRAS), which were published in 2011 [20]. While reporting the reliability and agreement of the study, the participant and observer populations, the process of assessment and data analysis, and the reliability and agreement of the reports have been described in detail [9].

### Study design

This was a cross-sectional study to assess the degree of inter-and intra-observer reliability and agreement. Three advanced trained raters (observers A, B, and C; observers A and B were blinded to the medical history of the infants with PWS) evaluated the same 15 GM video records of the infants by applying the “Assessment of Motor Repertoire-3 to 5 Months” and complying with the standardized assessment procedures [18].

### Participants

Fifteen infants with PWS were selected between November 2014 and December 2017 from the Rehabilitation Department of Children's Hospital of Fudan University. The study was given ethical approval by the Research Ethical Board of Children’s Hospital of Fudan University (Certificate number 2019(NO. 025)). Participants meeting the following inclusion criteria were included: (1) infants with definite PWS genetic diagnosis reports; (2) infants with at least one GM video recording that was carried out in the “Fidgety Movements” period; and (3) infants for whom written consent was provided by their legal guardian, allowing the video recordings to be used for research purposes. Participants who met the following exclusion criteria were excluded: (1) infants with brain MRI reports that showed severe brain injury lesions; and (2) infants who suffered from any other genetic metabolic disorders.

In all, there were 15 participants in this study, 12 of whom were male and 3 of whom were female. Birth weights ranged from 1802 to 3250 g, and the average weight was 2622.13 g. Gestational ages ranged from 33 to 38 weeks for3 infants and from 38 to 40 weeks for 12 infants, and the average gestational age was 38 weeks. PWS genetic diagnoses were confirmed between 3 and 65 weeks after birth, and the average age at the time of genetic diagnosis was 18.33 weeks. All 15 infants with PWS had received early intervention during the first four weeks of life. Early intervention programs included the following: Infants with PWS were assessed for sucking problems and failure to thrive. Developmental assessment was performed routinely. Physical and occupational therapies were performed to facilitate the development of motor milestones. Especially, feeding support and overcoming gravity training were emphasized during the first two months of life. Parents brought their infant to the hospital once a week for making an early intervention program which was performed at home every day and recorded the implementation in detail by parents. Each session lasts 10 to 15 min and the cumulative training time were more than 30 min a day [21–23]. Further details on the distribution of characteristics of the infants with PWS are outlined in Table 1.

**Table 1:** Characteristics of sample

Characteristic items	Statistical description
Gestational age (mean, SD) weeks	38, 1.81
Birth weight (mean, SD) grams	2622.13, 337.25
Age at fidgety assessment (mean, SD) weeks	12.73, 3.20
Age at PWS genetic diagnosis (mean, SD) weeks	18.33, 15.89
Early intervention	15(100%)
Male	12(80%)

### Observers

All three observers had completed the GM training courses and obtained the assessment qualification certificate offered by the GM Trust before starting this study [24]. They complied with the guidelines and grading criteria of the assessment of general movement and the “Assessment of Motor Repertoire- 3 to 5 Months” scales [18, 25, 26]. The three observers were assigned labels by the letters A to C; observers A and B were early intervention therapists for infants, and observer C was a rehabilitation doctor. All of them had rich experience in GM assessment in clinical practice, and observers A and B obtained advanced training course qualification certificates four years prior to the study. During the study period, observers A and B did not know the specific medical history and risk factors for the participants and were only provided with the ages of the infants at the time of video recordings. On the other hand, observer C was granted an advanced training course qualification certificate two year prior to the study. As the organizer of the present study, observer C knew the basic medical history of the participants but had no information on the previous GM assessment results.

### Video recordings

We searched GM videos by the ID numbers of the infants meeting the inclusion and exclusion criteria in the GMs application of diagnosis and treatment system databases of Children’s Hospital, Fudan University. GM videos were taken according to the guidelines outlined by Christa Einspieler [27]: (1) The infants were lying in the supine position with minimal clothing, no dummies (pacifiers) or toys, and in an adequate behavioural state. Sequences that included crying and fussing were discarded. (2) GM video recordings lasted for 3–5 min at a time. (3) GM videos were taken between 9 and 20 weeks of age (fidgety movements period) [28].

Accordingly, a total of 21 infant video records were retrieved. If a participant had more than one GM video record, one of the record was randomly selected as the research object. If there was only one GM video record for a participant, it was taken as the research object. Finally, 15 representative sequences of GM video records were included for the reliability and agreement study. All of the GM video recordings lasted between 3.1 min and 6.9 min, with an average time of 4.3 min.

### The assessment tool

The “Assessment of Motor Repertoire-3 to 5 Months” [16, 25] is an observational instrument designed to assess the GM video recordings of infants. It is divided into three main domains of observation, namely “Movement Patterns” (23 items), “Postural Patterns” (13 items), and “Movement Character” (8 items). The overall result (44 items) is taken as the basis for the “Motor Optimality List”, which is based on the scoring of five subcategories, the first of which rates “Fidgety Movements” as normal (12 points), abnormal (4 points) or absent (1 point); the second subcategory, “Repertoire of Co-Existent Other Movements”, is classified as age-adequate (4 points), reduced (2 points) or absent (1 point); the third subcategory, “Quality of Other Movements”, is evaluated by the number of normal or abnormal items within the “Movement Patterns” field: a number of normal patterns (N) higher than that of abnormal patterns (A) scores 4 points; *N* = A scores 2 points; and *N* < A scores 1 point. The fourth subcategory, “Posture”, is assessed in the same way, based on the items of the second main field of observation, “Postural Pattern”. The fifth subcategory, “Movement Character”, describes the overall movement character observed in all movement categories: smooth and fluent (4 points); abnormal, but not cramped-synchronised (2 points); and abnormal and cramped-synchronised (1 point). Finally, the scores of each subcategory are added, resulting in a total MOS of 5 to 28 points. The higher the score, the stronger the motor repertoire [18, 19, 26, 28].

### Assessment procedure

Observers assessed the 15 GM video records in independent locations by using computers. Observers were allowed to watch the videos repeatedly but were not allowed to communicate with each other. The score sheet was numbered consecutively from 1 to 15 depending on the birth date of the infants, and the video records were assessed in a prearranged order [27, 28].

Each observer completed the assessment of the GM video records on the same day as the inter-observer reliability part of the study. However, the time interval between the two assessment time points of the same GM video records for the same observer was required to be more than three months in the intra-observer (re-test) reliability part of the study.

### Statistics

All data were analysed by statisticians using the SPSS (Statistical Product and Service Solutions) (version 17.0, SPSS Inc., Chicago, Illinois, United States). The respective subcategory agreement in the “Assessment of Motor Repertoire—3 to 5 Months” was identified by means of kappa statistics or expressed in terms of percent agreement if the kappa value could not be determined. The statistical measure of the Cohen's kappa was used to determine intra- and inter-observer agreement, considering the agreement by chance [19]. The interpretation of the results complied with the guidelines by Landis and Koch [29], who classify a κ value of < 0.20 as poor agreement, 0.21- 0.40 as fair, 0.41- 0.60 as moderate, 0.61- 0.80 as good, and 0.81–1.00 as very good agreement. Intraclass correlation coefficient (ICC) statistics were applied to examine pairwise agreement of the sum scores among the observers in the “Assessment of Motor Repertoire- 3 to 5 Months”. ICCs are correlation coefficients that allow comparison of two or more repeated measurements [29]. For the “Motor Optimality Score”, ICC statistics were applied to examine pairwise intra- and inter-observer agreement (A-B, A-C, B-C, A-B-C) and agreement among all three observers (A-B-C). The measurement error was termed “Sw”, and it was calculated as the square root of the mean within-subject variance. The 95% confidence intervals (95% CI_s_) for the correlation coefficients were also analysed [30].

## Results

### Intra- and inter- observer agreement of sum scores among the observers in the MOS

As expected, the infants who that participated in the study received scores ranging in the lower part of the 5- to 28-point total MOS. Intra- and inter- observer agreement for the total MOS were reported.

In accordance with the ICC values, as shown in Table 2 (inter- observer agreement) and Table 3 (intra- observer agreement). The ICC values ranged between 0.86 and 0.95 for pairwise inter- observer agreement. The overall inter-observer agreement was 0.93. Meanwhile, regarding pairwise intra- observer agreement, the ICC values ranged between 0.95 and 0.98.

**Table 2:** Inter-tester reliability of the total “Motor Optimality Score” pair wise and between the three observers (A-C), comparing Intraclass Correlation Coefficient (ICC), 95% confidence intervals (95% CI), and within subject standard deviation (Sw)

Observers	ICC	95%CI	Sw
A-B	0.86	0.58–0.95	2.54
A-C	0.95	0.85–0.98	2.64
B-C	0.89	0.67–0.96	2.66
A-B-C	0.93	0.84–0.98	3.82

*N* = 15 observations for A, B and C

**Table 3:** Intra-tester reliability of the total “Motor Optimality Score” pair wise and between the three observers (A, B, C), comparing Intraclass Correlation Coefficient (ICC), 95% confidence intervals (95% CI), and within subject standard deviation (Sw)

Observers	ICC	95%CI	Sw
A	0.98	0.94–0.99	2.58
B	0.95	0.85–0.98	2.90
C	0.95	0.86–0.98	2.74

*N* = 15 observations for A, B and C

The measurement error (Sw) ranged from 2.54 to 2.66 between the various pairs of observers in the assessment of the MOS. The overall Sw was 3.82 between the observers. Sw ranged from 2.58 to 2.90 between the same pairs of observers before and after the assessment of the MOS.

### Intra- and inter- observer agreement of scores in the respective subcategories among the observers

The assessment results for the subcategories of “Fidgety Movements” and “Movement Character” achieved complete agreement among the three observers (A-C), in which the “Fidgety Movements” total was absent (1 point) and the “Movement Character” total was abnormal, but not cramped-synchronised (2 points). Consequently, no kappa value for “Fidgety movements” and “Movement Character” could be calculated among the three observers (A-C). Therefore, agreement between the observers (A-B, A-C, and B-C) regarding the subcategories “Fidgety Movements” and “Movement Character” were expressed in terms of percent (100%) (Table 4).

**Table 4:** Inter-tester reliability of “Assessment of Motor Repertoire-3 to 5 Months” subcategories

Subcategories	Tester A-B	Tester A-C	Tester B–C
	κ (se κ)^a^ or	κ (se κ)^a^ or	κ (se κ)^a^ or
	%	%	%
Fidgety	100%	100%	100%
Repertoire	0.63(0.32)	0.63(0.32)	1.00(0.00)
Quality	0.77(0.14)	0.66(0.52)	0.66(0.16)
Posture	1.00(0.00)	1.00(0.00)	1.00(0.00)
Character	100%	100%	100%

Pair wise analysis between the observers (A-C) based on video recordings of 15 infants, expressed in kappa (κ)-values or percent (%) agreement

^a^se(κ) = standard error of κ

In the other subcategories, data from all 15 infants were included in the analysis. Moderate to high inter-observer reliability was achieved in the assessment of “Repertoire of Co-Existent Other Movements” and “Quality of Other Movements”, with kappa values ranging between 0.63 and 1.00, and one single value reached perfect agreement in the “Repertoire of Co-existent Other Movements” (B-C) category. The assessment of “Posture” resulted in very good kappa values in pairwise inter- observer agreement (A-B, A-C, B-C), where the total kappa value totals was 1.00 (Table 4).

For the Intra-observer agreement (A, B, C) between the respective subcategories, data from all 15 infants were included in the analysis. The subcategories “Fidgety Movements,” “Movement Character” (A, B, C), and “Posture” (B) were expressed in terms of percent (100%). In the other subcategories, moderate to high intra-observer reliability was achieved in the assessment of the “Repertoire of Co-Existent Other Movements” (A, B, C), “Quality of Other Movements” (A, B, C), and “Posture” (A, C) subcategories, with kappa values ranging between 0.63 and 1.00 (Table 5).

**Table 5:** Intra-tester reliability of “Assessment of Motor Repertoire-3 to 5 Months” subcategories

Observers	Fidgety	Repertoire	Quality	Posture	Character
	κ (se κ)^a^ or	κ (se κ)^a^ or	κ (se κ)^a^ or	κ (se κ)^a^ or	κ (se κ)^a^ or
	%	%	%	%	%
**A**	100%	0.63(0.32)	0.73(0.17)	1.00(0.00)	100%
**B**	100%	0.63(0.32)	0.67(0.16)	100%	100%
**C**	100%	1.00(0.00)	0.67(0.15)	0.64(0.31)	100%

Pair wise analysis between the three observers (A-C) based on video recordings of 15 infants, expressed in kappa (κ)-values or percent (%) agreement

^a^se(κ) = standard error of κ

## Discussion

The clinical phenotypes of PWS vary greatly with age. During the early period after birth, motor performance is particularly affected in infants with PWS. Most infants present as severely hypotonic, inactive and sometimes almost motionless in the neonatal period. Despite the fact that they persistently suffer from hypotonia, muscle weakness, and severe motor development delays, young infants with PWS gradually become more responsive and present more spontaneous movements weeks or months later [6, 31]. Motor problems occur after birth and continue into adulthood. PWS patients score well below the normal standard range on standardized motor performance tests [32]. Holm VA was the first to propose the PWS clinical scoring diagnosis system based on the characteristics of the clinical symptoms in 1993. This system subdivided and summarized each clinical manifestation by score according to age, realizing the clinical standardized diagnosis of PWS. For infants with obvious hypotonia, difficulty feeding and gonadal dysplasia, the possibility of PWS should be suspected [33]. However, in terms of the method of carrying out a standardized quantitative assessment of motor performance, particularly in individuals with suspected PWS or infants who were diagnosed with PWS at a young age (less than 6 months old), there is still a lack of relevant research in this field.

It is extremely difficult to carry out prospective behavioural analyses for rare disorders [11]. However, GM assessment (video recording assessments) has been applied for the early screening and assessment of genetic metabolic diseases [34]. Christa Einspieler and her colleagues conducted a longitudinal study on the predictive value of movements and postures on the development of neurological deficits later in life for infants with Smith-Magenis syndrome. The findings, which included significantly reduced motor repertoire, absent fidgety general movements, abnormal posture, and jerky and monotonous overall movements, indicated severe motor impairment as early as 4 months of age [11]. Similar results have been shown for other genetic metabolic disorders, where the infants with the most severe phenotypes show no fidgety movements at aged of 3–5 months, while their neurodevelopmental status was found to be more or less normal [12, 35, 36]. Until now, there has been little systematic data on the early neurodevelopmental functioning of infants with PWS, and this study will be the basis for preliminary research to document a behavioural manifestation of the syndrome in infants as young as 4 months of age by the “Assessment of Motor Repertoire -3 to 5 Months”. The absence of fidgety movements and the presence of characteristic clinical phenotype indicate a risk of maldevelopment. Therefore, genetic testing and early intervention are reasonable recommendations. Early intervention plays a positive role in promoting infant development and improving developmental outcomes.

In this reliability study of the detailed general movements assessment (Assessment of Motor Repertoire-3 to 5 Months), we obtained the following results: (1) Three observers who were qualified members of the GM Trust participated in the assessment of video recordings of spontaneous movements for infants with PWS. The overall reliability ICC values of the MOS ranged from 0.84 to 0.98 for the three observers, which indicated that the agreement reliability among the raters reached a very high degree. The ICC values for the MOS ranged between 0.86 and 0.95 for pairwise agreement in inter- observer reliability, where the highest ICC value was 0.95(95% CI 0.85–0.98) for A-C, followed the ICC value of 0.89(95% CI 0.67–0.96) for B-C, and the ICC value of 0.86(95% CI 0.58–0.95) for A-B. The ICC values ranged between 0.95 and 0.98 for the Intra-tester reliability of the MOS between the first and the second rating time-points of the observers. Therefore, in the PWS population, the MOS has very good interobserver reliability and retest reliability, which can be applied in the clinical quantitative evaluation of early motor performance for infants with PWS. (2) The subcategory “Fidgety Movements” in the “Assessment of Motor Repertoire-3 to 5 Months” was divided into three subtypes: normal fidgety movements (scores of 12), abnormal fidgety movements (scores of 4) and absent fidgety movements (scores of 1). The assessment results of all observers (A-C) were completely consistent for thoes of absent fidgety movements(scores of 1)based on the severely hypotonic, inactive, and sometimes almost motionless infants with PWS in the neonatal period seen in this study. Because Kappa statistical analysis is based on multicategorical variables, the Kappa valuecould not be calculated with the single variable for the outcome of absent fidgety movements in this study. Therefore, the percentage of agreement represented the detailed impression of the degree of the reliability and agreement in the subcategory “Fidgety Movements”. Complete agreement reliability (100%) was achieved for both the interobserver reliability (A-B, A-C, B-C) and the re-test reliability of the observers (A-C) in this study. Inter-and intra-observer reliability in the “Movement Character”subcategory in the “Assessment of Motor Repertoire-3 to 5 Months” was the same as the reliability of the “Fidgety Movements” category. (3) The points achieved in the subcategories”Quality of Other Movements”and “Posture” were calculated based on the sum of the number of normal and abnormal items in their respective category entries. Accordingly, the result was not simply based on the inter- and intra-observer agreement for each item, even if the agreement reliability values for each item of these subcategories were determined to be low, which did not necessarily affect the MOS or the ICC values of reliability. Moderate to high inter-observer reliability was achieved in the assessment of “Quality of Other Movements” among the observers (A-B, A-C, B-C), with kappa values ranging between 0.66 and 0.77. The assessment of “Posture” resulted in very good kappa values in pairwise inter- observer agreement (A-B, A-C, B-C), and the total kappa value was 1.00. Moderate to high intra-observer reliability was achieved in the assessment of “Quality of Other Movements” (A, B, C), and “Posture” (A, C), with kappa values ranging between 0.64 and 1.00. Complete agreement reliability (100%) was achieved in the re-test reliability of “Posture” for observer B.

Compared with previous studies of genetic metabolic disorders, the reliability study of detailed GM assessments for inter-observer agreement for infants with Smith-Magenis syndrome indicated Kappa values between 0.82 (assessment of posture) and 1.00 (assessment of fidgety movements) and is similar to the research results of the two parts of this study [34]. In the reliability study of high-risk infants, inter-observer reliability in the assessment of the total MOS was high between observers as ICC was the 0.87, and the ICCs for the pairwise analyses ranged between 0.80 and 0.94, which is consistent with the results of this study [19]. However, the agreement reliability regarding the subcategories “Posture” and “Movement Character” in this study was better than that for infants with a high risk of brain injury, in which the “Movement Character” kappa value was 0.54–0.84 and the “Posture” kappa value was 0.39–0.56.

In this study, the “Assessment of Motor Repertoire-3 to 5 Months” in infants with PWS had high in inter-and intra-observer reliability, which was not only related to the characteristics of early motor performance in the infants with PWS but also had a great correlation with the following factors: (1)The video acquisition process requires strict regulations to ensure the high quality assessment of video recordings, which plays an important role in the accuracy of GM assessment results based on Gestalt perception theory. (2) Three observers completed the advanced training courses for GMs and had rich experience in GM assessment in clinical practice. In addition, we held a GM assessment quality control meeting once a week to discuss difficult GM cases and the standardized assessment process. All of these clinical practices help to greatly improve the accuracy and consistency of the GMs assessment results among observers.

Of course, there were also some limitations in this study. On the one hand, the population characteristics of infants with PWS were less representative in this study. More than 90% of the infants with PWS had a “Total Optimality Score” under 9 points, and the sample size was also very limited in this study. On the other hand, the assessment results were completely consistent for absent fidgety movements(scores of 1)for the infants with PWS in this study. Thus, the assessment of “Fidgety Movements”, which itself showed good inter-observer agreement, had a significant effect on the ICCs for the total MOS. Therefore, the sample size and study units should be increased in later studies to reduce research bias.

## Conclusion

To our knowledge, this is the first report on clinical cross-sectional study of GMs assessment in patients with PWS. This study especially focused on the inter-and intra-observer reliability of the detailed GMs assessment which is the “Assessment of Motor Repertoire-3 to 5 Months” in infants with PWS. Particularly, the “Motor Optimality Score”, the subcategory of “Fidgety Movements”, “Movement Character” and “posture” were expressed with perfect agreement in inter- observer and re-test reliability. This study provides a basis for further analysis of the characteristics and correlation among PWS, cerebral palsy and normal infants in early performance movements based on the “Assessment of Motor Repertoire-3 to 5 Months”.

## Data Availability

The datasets generated and/or analysed during the current study are not publicly available due which was just a small part of another study which is in process but are available from the corresponding author on reasonable request.

## Abbreviations

GMA: General Movement Assessment
GMs: General Movements
MOS: Motor Optimality Score
ICC: Intraclass Correlation Coefficient
PWS: Prader-Willi Syndrome
